# Supplementary material for: The Roles of TP53 and FGFR2 in Progress Made Treating Endometrial Cancer
Source: Diagnostics (Basel). 2022 Jul 18;12(7):1737. doi: 10.3390/diagnostics12071737 (PMC9316851; doi:10.3390/diagnostics12071737)
Supplement: Supplementary file 1 [file diagnostics-12-01737-s001.zip › diagnostics-1741876-supplementary.pdf]

Table S1. AmpliSeq for Illumina Cancer Hotspot Panel v2 Genes.

BL1	EGFR	GNAS	KRAS	PTPN11
AKT1	ERBB2	GNAQ	MET	RB1
ALK	ERBB4	HNF1A	MLH1	RET
APC	EZH2	HRAS	MPL	SMAD4
ATM	FBXW7	IDH1	NOTCH1	SMARCB1
BRAF	FGFR1	JAK2	NPM1	SMO
CDH1	FGFR2	JAK3	NRAS	SRC
CDKN2A	FGFR3	IDH2	PDGFRA	STK11
CSF1R	FLT3	KDR	PIK3CA	TP53
CTNNB1	GNA11	KIT	PTEN	VHL

Table S2. Detailed characterization of the studied cohort. OS, RFS.

	**N**	**Average**	**Median**	**SD**	**Minimum**	**Maximum**
Age (years)						
< 50	2	49	49	1,41	48	50
51-60	16	56,69	57	2,70	52	60
61-70	34	65,59	65,5	2,88	61	70
71-80	30	75,43	75	3,17	71	80
81-90	15	83,93	83	2,22	81	89
> 90	6	94	93,5	2,68	91	99
Total	103	71,08	70	10,98	48,00	99,00
BMI						
18,5-24,99	2	23,9	23,9	1,27	23	24,8
25-29,99	1	29,8	29,8	-	29,8	29,8
30-34,99	61	33,06	33	1,20	30	34,9
35-39,99	39	36,29	36,2	1,01	35	38,5
Total	103	34,07	34,20	2,44	23,00	38,50
Rtg-therapy	59	4938,98	4600,00	1132,59	4500,00	9200,00
OS (years)	102	8,23	9,00	4,42	1,00	15,00
RFS(years)	6	4,67	3,00	4,23	1,00	10,00

Table S3. Histopathological types of EC.

	**Total**	**G1**	**G2**	**G3**
Endometriod adenocarcinoma	95	49	39	7
Serous adenocarcinoma	5	1	2	2
Adenosquamous carcinoma	1	-	-	1
Clear cell adenocarcinoma	1	-	-	1
Mixed endometroid and clear cell carcinoma	1	-	-	1

Table S4. The mutation spectrum according to histopathological type and grade. Moreover, there is presented an impact of mutated genes on TNM, OS, and RFS.

Gene	AKT1	APC	ATM	CTNNB1	ERBB2	FBXW7	FGFR2	KRAS	No mutation in	PIK3CA	PTEN	PTPN11	RB1	RET	TP53

Endometrioid carcinoma															
G1			1	3		1	1	9	10	10	16	2	1	1	3
G2	1	1		5	1	5	7	7		11	15				8
G3							1	1		1	1				2
G progress							$p < 0.01$								$p < 0.01$
Clear cell carcinoma									1						
Carcinosarcoma								1							1
Clinical meaning and type of mutation															
TNM progress							$P < 0.01$								
TNM vs number of mutation															$p < 0.01$
Age								$p > 0.05$							
RFS and OS								$p > 0.05$							
